# Serologic response to pneumococcal vaccination in children experiencing recurrent invasive pneumococcal disease

**DOI:** 10.1186/s12879-018-3267-6

**Published:** 2018-08-06

**Authors:** Helene A. S. Ingels, Bjørn Kantsø, Hans-Christian Slotved

**Affiliations:** 1 Department of Pediatrics, Slagelse Hospital, Slagelse, Denmark; 2 Department of Bacteria, Parasites and Fungi, Statens Serum Institut, Artillerivej 5, 2300 Copenhagen, DK Denmark; 3 Department of Microbiological Diagnostics &Virology, Statens Serum Institut, Copenhagen, Denmark

**Keywords:** *Streptococcus pneumoniae*, Vaccination, Recurrent disease, Non-responder, Serology, Recurrent IPD

## Abstract

**Background:**

Some children are prone to recurrent invasive pneumococcal disease (rIPD) and of these, some respond insufficiently to standard pneumococcal vaccination. Little is known about how to handle these children and if they benefit from additional vaccination. Here, we present results from a nationwide study of pediatric rIPD including data on serotype-specific vaccination response to pneumococcal polysaccharide vaccination (PPV23) and pneumococcal conjugate vaccination (PCV7/13).

**Methods:**

A retrospective, population-based study was conducted using The National *Streptococcus pneumoniae* Registry, which contains laboratory-confirmed data from all cases of IPD in Denmark. From January 1980–June 2013 all children aged 0–15 years with rIPD were identified. Clinical data and data on serotype-specific pneumococcal antibody response were collected. Over the years quantification of pneumococcal antibodies varied from being presented in arbitrary units (ELISA), in μg/ml (WHO ELISA) and lately in μg/ml based on Luminex technology.

**Results:**

2482 children were diagnosed with IPD and 75 episodes of rIPD were documented in 59 children. An underlying disease was documented in 45 (76%) children. Vaccination data were available for 26 children; 11 were vaccinated solely with PPV23, 8 with a combination of PPV23 + PCV7, 5 with PCV7 and 2 with PCV13. In total, nine responded to PPV23 vaccination and ten were PPV23 non-responders. Of the 15 PCV vaccinated children, two children responded subnormal to PCV7. Among PPV23 non-responders, five responded to subsequent PCV vaccination.

**Conclusions:**

In our population-based study of children with rIPD 53% of the children responded insufficiently to PPV23 vaccination. PPV23 non-responders benefitted from PCV vaccination.

**Electronic supplementary material:**

The online version of this article (10.1186/s12879-018-3267-6) contains supplementary material, which is available to authorized users.

## Background

Invasive pneumococcal disease (IPD) causes significant morbidity and mortality in children [1]. Primarily children with known risk factors experience recurrent IPD (rIPD). Between 40—92% of children with rIPD have comorbidities such as leukemia, cerebrospinal fluid leak or primary immune deficiency [2–8]. Some children with rIPD respond insufficiently to pneumococcal vaccination [9]. Little is known about how to handle these children and if they benefit from additional vaccination.

The polysaccharide vaccines (PPV14 and later the PPV23) have been available since the late 1970s. They are T-cell independent vaccines and therefore not immunogenic in children under the age of 2 years, who suffer the highest burden of disease. Vaccination with the pneumococcal conjugate vaccines (PCVs) has been possible since 2000. The conjugation of polysaccharides to a carrier protein results in a T-cell dependent immune response and induction of memory cells and thereby the possibility for a booster response upon subsequent antigen exposure [10–12].

The introduction of conjugate vaccines in childhood vaccination programmes has had a significant impact on the morbidity caused by *Streptococcus pneumoniae*, including in Denmark [13, 14]. However, IPD does still occur and it is important to protect children at risk of IPD. Schedules combining PCVs with PPV23 have been proposed and studied in order to expand disease protection against serotypes not included in the PCVs [11, 15, 16], but little is known on vaccination response in children with rIPD.

In this paper we present results from a 33-year retrospective nationwide study of an unselected population of children with rIPD. We present data on vaccination response in 26 children experiencing rIPD. The specific aim was to quantify the serotype specific vaccination response to PPV23 and PCV in children experiencing rIPD and to discuss the clinical management of these children.

## Methods

### Study design

A retrospective, nationwide analysis was conducted using The National *S. pneumoniae* Registry, which contains laboratory-confirmed data from IPD cases in Denmark dating back to 1938. Pneumococcal isolates have been submitted from all Departments of Clinical Microbiology in Denmark on a voluntary basis (since 2007 mandatory [17]) to the National Reference Center at Statens Serum Institut. It has been estimated that more than 90% of all IPD isolates are submitted to the reference laboratory [18].

**Fig. 1:**
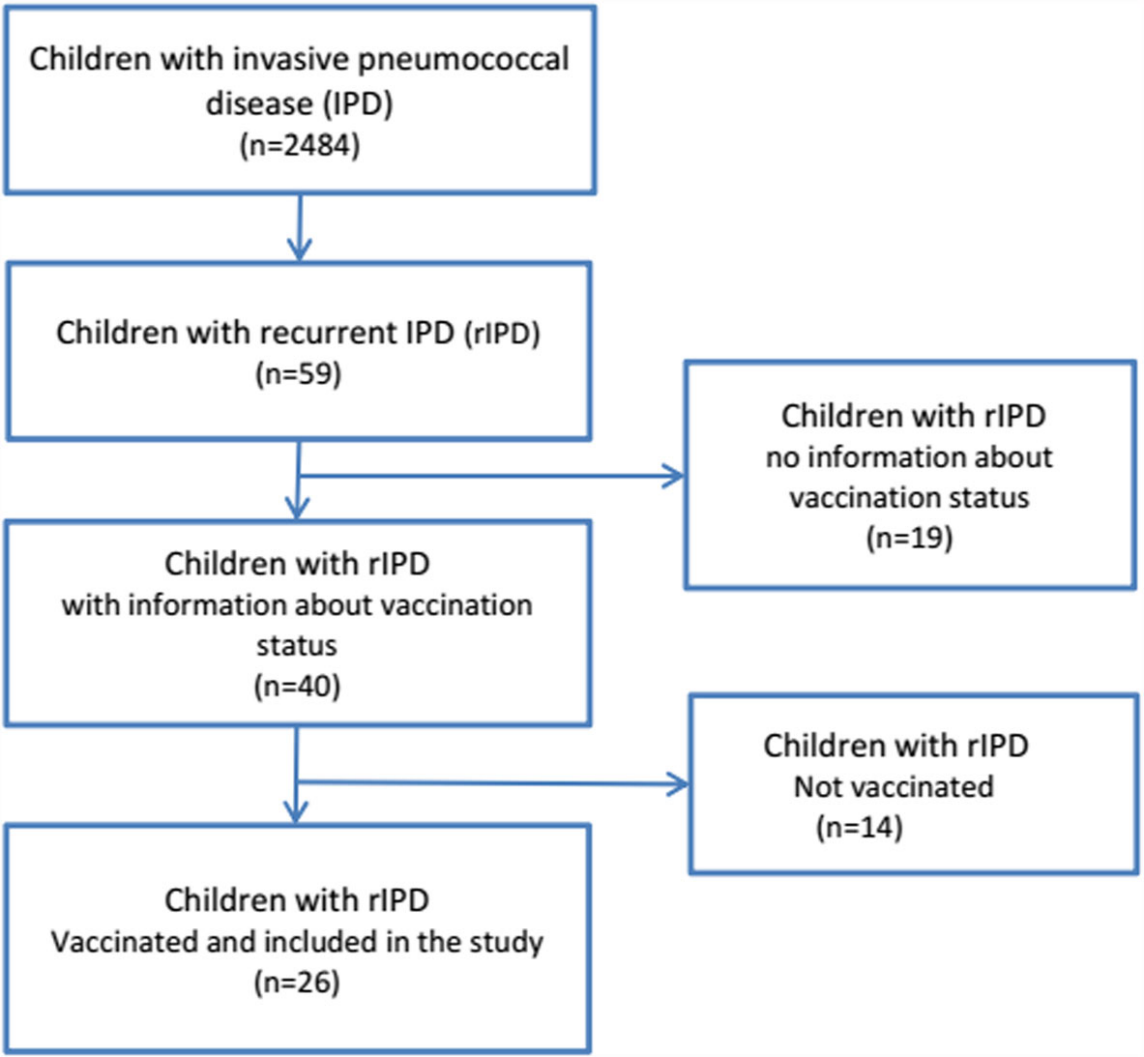
Inclusion of children with recurrent invasive pneumococcal disease

From this registry all children aged 0–15 years with laboratory-confirmed IPD from January 1980 through June 2013 were identified and children with recurrent IPD (rIPD) in the period were included in the study (Fig. 1), as previously described [7]. Recurrent episodes were defined as isolation of *S. pneumoniae* from any normally sterile site ≥30 days after an initial positive culture or ≤ 30 days if the recurrent infection was with a new pneumococcal serotype. Medical records of children experiencing rIPD were reviewed to identify date and type of pneumococcal vaccination, date of evaluation of serological pneumococcal antibody (PNA) response. Demographic, clinical and microbiologic data were obtained.

### Serotype specific antibody measurement

Data on PNA response were obtained from the medical journals and verified in the national serological laboratory at Statens Serum Institut, Copenhagen. During the study period from 1980 until 2013, quantification of pneumococcal antibodies varied between different methods

A. Arbitrary units based on a ELISA procedure [19, 20].
B. In μg/ml (WHO/ELISA). This was an update of the ELISA procedure according to the WHO guidelines and included adsorption with both CWPS and 22F polysaccharide [21].
C. Since 2011 in μg/ml based on Luminex technology using CWPS and 22F adsorbtion as described in details elsewhere [22–24].

### Definition of PPV23 response

#### PNA measured in arbitrary units

A non-responder was a child over 2 years of age with a geometric mean antibody titer below 25 units or below 40 units with more than one of the six type specific antibody titer below 25 units. A responder was defined as having a response above the mentioned criteria. More specific details on the used method, calculation of the arbitrary units, and definition of a non-responder vs. responder are outlined in the following studies [19, 20].

#### PNA measured in μg/ml

A child over 2 years of age with a response (over threshold 1.3 μg/ml) to half or more of the six-twelve tested serotypes was defined as being a PPV23 responder. Children with an impaired response to more than half of the tested serotypes were defined as being non-responders according to most common international standards [25–27]. In cases where children were vaccinated with first the PCV and subsequently the PPV23 the child could only be classified as a PPV23 reponder after evaluation of the serotypespcific response to non PCV serotypes.

### Definition of PCV response

A responder to PCV was defined as a child having a response of 0.35 μg/ml or more for each of the tested serotypes [12].

#### Pneumococcal vaccination in Denmark

In Denmark, routine infant pneumococcal vaccination with PCV7 (Prevenar 7, Pfizer) was implemented in 2007 [17]. Since 2001 PCV7 was recommended for use in certain pediatric risk groups [28]. PCV7 was replaced by the PCV13 from April 2010 [13]. Since the early 1980s a 23-valent polysaccharide vaccine (Pneumova× 23(PPV23), Merck) have been licensed and the vaccine are used in high-risk children above two years and adult risk groups [29].

#### Ethical approval

The Danish Data Protection Authority (Datatilsynet) has approved usage of the data (j.nr 2013–41-2568).

## Results

### Epidemiology and clinical characteristics of cohort

During the 33-years study period 2482 children were diagnosed with IPD. Among the 2418 children surviving their first episode of IPD for more than 30 days, 75 episodes of rIPD were documented in 59 children. Among the 59 children, 76% had a predisposing underlying disease at the time of the rIPD, the most common conditions were immune deficiency due to transplantation, anatomic abnormalities or complement deficiency (more details described in Ingels et al. [7, 9]).

Of the 59 children, vaccination data were available for 68% (n: 40) of the cohort; 14 received no vaccination and 26 children were vaccinated (Fig. 1). Data on pneumococcal antibody response were available in all vaccinated children.

Among the 26 children who where vaccinated, five children were vaccinated solely with PCV7, two children with PCV13, eight with a combination of PPV23 and PCV7/13 and eleven of the children were vaccinated with PPV23. Table 1 presents specific vaccination data.

**Table 1:** Vaccination Response of children experiencing repetitive episodes of invasive pneumococcal disease

Patient	Underlying disease	Localization of Infection (serotypes)	Vaccine (s) given	Responder (R) / Non Responder (NR)
1	Complement C2 deficiency	Bacteremia × 2 (**6B**/34)	PPV23	(NR)
2	Complement C2 deficiency	Bacteremia × 2 (**12F/4**)	PPV23	(NR)
3	Syndrome, undefined	Bacteremia × 2 (**7F/23F**)	PPV23 multiple	(NR)
4	Shunt (Dandy-Walker syndrome)	Bacteremia × 2 (**19F/19F**)	PPV23 × 2	(NR)
5	HSCT (MB Gaucher)	Bacteremia × 2 (**18C/18C**)	PPV23	(NR)
6	No underlying condition found	Bacteremia × 2 (**6B/6B**)	PCV7 × 2, PPV23	PCV7 (R), PPV23 (NR)
7	No underlying condition found	Bacteremia × 2 (**6B/6B**)	PCV7 × 3, PPV23	PCV7 (Subnormal (Table 2), PPV23 (NR)
8	No underlying condition found	Bacteremia × 2 (**6A**/24F)	PCV13, PPV23 × 2	PCV13 (R), PPV23 (NR)
9	Complement C2 deficiency	Bacteremia × 3 (**6B**/16F/**14**)	PCV/ × 2, PPV23	PCV7 (R), PPV23 (NR)
10	Syndrome with malabsorption	Bacteremia × 3 (**14/19A/19A**)	PCV7, PPV23	PCV7 (R), PPV23 (NR)
11	Toll-Like receptor signaling defect	Bacteremia × 5 (15C/7/7/38/NTpn)	PCV7 × 3	PCV7 (Subnormal) (Table 2)
12	Leukemia	Bacteremia × 4 (**18C/8/8/8**)	PCV7 × 2, PPV23	R
13	No underlying condition found	Meningitis × 2 (**14/6B**)	PCV7 × 3	R
14	Univentricular heart	Bacteremia × 2 (**33F/33F**)	PPV23	R
15	Complement C2 deficiency	Meningitis × 2 (**4/14**)	PPV23 x multiple	R
16	Klippel Trenaunay syndrome	Bacteremia × 3 (6A/24F/24F)	PPV23	R
17	Asplenia congenitalis	Meningitis × 2 (**6B**/6A)	PPV23	R
18	Complement C2 deficiency	Bacteremia × 2 (**9 V**/NTpn)	PCV7 × 2, PPV23	R
19	No underlying condition found	Meningitis/bacteremia (**19F**/1)	PCV7 × 2	R
20	Hepatic disease (Biliary atresia)	Bacteremia × 2 (9 N/23A)	PCV7 × 2	R
21	CNS tumor, chemo treatment	Bacteremia × 2 (**9 V/1**)	PCV7 × 2	R
22	HSCT (myelodysplasia)	Bacteremia × 2 (**6B/6B**)	PCV23	R
23	Complement C2 deficiency	Bacteremia × 2 (**6B/**38)	PPV23	R
24	Asplenia congenitalis	Meningitis × 2 (**23F**/15C)	PCV7 × 2, PPV23	R
25	No underlying condition found	Bacteremia × 2 (**19A/19A**)	PCV13	R
26	Hepatic disease (Biliary atresia)	Bacteremia × 2 (9 N/23A)	PCV7	R

*HSCT* Haematopoietic Stem Cell Transplantation, *PCV* Pneumococcal conjugate vaccine, *PPV* Pneumococcal polysaccharide vaccine, *NTPn* Non typeable *S.pneumococcus,* Serotypes in bold are included in the vaccine (s) given to the particular child

#### Vaccination response to PCV7/13

In total, 15 of the children exeriencing rIPD were vaccinated with the PCV7 or PCV13. Of these, two were vaccinated as a result of the general infant PCV vaccination program [16] and 13 were offered PCV as part of the recommendations for vaccination of high-risk children [27]. All except two children (pt 7 and pt. 11) responded sufficiently to all of the tested serotypes (Table 1). Patient 7 experienced rIPD and septicemia with *Kingella kingae* in the first three years of life, but after the age of five no enhanced susceptibility to infections could be documented. After three doses of PCV7 he still responded subnormally with a response to four of seven serotypes (Table 2). Patient 7 mounted a normal response to serotype 6B eventhough this was the serotype he was infected with twice. In contrast, immunoglobulins were in normal ranges and functional antibody response to Diphtheria-, Tetanus-, *Haemophilus influenzae* type b- and Polio vaccine were normal. This boy was a non-responder to PPV23 as well and was subsequently diagnosed with specific antibody deficiency toward pneumococcal polysaccharides (SAD) [25, 29]. Patient 11, a boy who experienced five times IPD, was diagnosed with a toll-like receptor (TLR) signaling deficiency [29]. This child had a subnormal PCV7 response with a response under threshold in four of the serotypes in spite of repetitive PCV vaccination (Table 2). The immunoglobulins of this child were in normal ranges, but responses to Diphtheria, Tetanus and *Haemophilus influenzae* type b vaccine were impaired as well.

**Table 2:** Subnormal Vaccination Response to PCV7 in two Children with rIPD

Serotype		6B	4	9 V	14	18C	19F	23F
Serotypespecific response (ug/ml)	Patient 7	0.80	*0.21*	0.56	0.63	*0.17*	0.97	*0.34*
	Patient 11	0.41	*0.24*	*0.12*	0.49	*0.05*	0.42	0.5

Numbers in Cursiva: serotypespecific response below threshold (0.35 μg/ml) (ref 28)

### Vaccination response to PPV23

Overall 19 children were vaccinated with PPV23. Of these, nine (47%) responded PPV23 and 10 (53%) were non-responders. Among PPV23 non-responders, five children were vaccinated with PCV7/13 at least twice and they all mounted a PNA response over threshold (pt 6—10 Table 1).

Comparison between PPV23 responders and non-responders revealed no differences regarding sex or age. Among PPV23 non responders, three of 10 had no known underlying condition at time of vaccination, thereby fulfilling the criteria of SAD ([29]), whereas in the PPV23 responding group all children had well defined underlying diseases such as asplenia congenitalis and leukemia. Among the six complement deficient children in our cohort, three responded to PPV23 and three were PPV23 non-responders (more details are presented in [9]).

#### Serotype specific response

For both the PCV7 and the PPV23 we observed serotypes where the patients could not mount an acceptable response. For the PCV7, children were most likely to respond to 6B, 19 V, 14 and, 19F, whereas antigens to 4, 18C and 23F in some children did not result in a response over threshold. For PPV23 we observed a low response for serotype 1, 7F and 18C (Additional file 1: Table S1).

## Discussion

In this nationwide study of pediatric rIPD, we found that, two children failed to respond acceptably to the PCV7. Moreover, more than half of the children vaccinated with PPV23 had a reduced vaccination response. The group was heterogenous and no clear differences were observed regarding background disease between PPV23 responders and PPV23 non responders.

Our results illustrate the importance of evaluating pneumococcal antibody response on an individual level in children at high risk of experiencing IPD. The identification of vaccine non-responders is important and may allow optimization of vaccination status and consideration of other prophylactic measures, such as treatment with replacement immunoglobulin, prophylactic antibiotics and information to the parents and the child about how to handle signs of infection*.*

Most PPV23 non- responders had a known background disease, however three of the non-responders occurred in apparently healthy children. Antibody response to polysaccharide antigens is impaired in young children less than two years of age. If this non-responsiveness to polysaccharides persist over the year of two it becomes an abnormality. In our cohort three apparently normal children over the age of two failed to respond to PPV23, fulfilling the criteria of Specific Antibody Deficiency (SAD). Nonresponsiveness to pneumococcal polysaccharides may be the only detectable immune abnormality or it may be associated with IgG2 subclass deficiency or other B-cell disturbances [26, 27]. The prevalence of this selective immunodeficiency is not known, but the condition is a common finding in some groups of children [25–27]. SAD can be associated with repetitive upper respiratory tract infections [25], which may lead to invasive disease. Susceptibility to infections in these children may decrease over time, although in some children the condition progresses to Common Variable Immunodeficiency (CVID) [30, 31], which is the reason that these children may require long-term follow-up.

We documented two children with a subnormal PCV response. Hyporesponsiveness to a single or two PCV antigens in children has previously been described [32–35]. It has been suggested that serotype-specific hyporesponsiveness in apparently healthy children may be a result of nasopharyngeal carriage of that particular pneumococcal serotype at the time of vaccination [33]. Moreover it is wellrecognized that not all serotypes in the PCVs are equally immunogenic. [12, 34, 36] and that the immune response can vary according to the number of doses administered [36]. A way to handle children lacking an antibody response to all PCV serotypes is to revaccinate with an extra PCV dose, which may lead to a sufficient response [37]. A child with a subnormal response for several PCV serotypes, like in our two patients, is an unusual clinical situation and suggests severe B-cell defect and/or underlying T-cell dysfunction. [37]. The compromised vaccination response of the child with TLR deficiency is in accordance with observations from others [38, 39]. Interestingly, one of our patients who responded subnormally to PCV experienced two episodes with IPD due to serotype 6B episodes but was putatively protected against 6B according to the serology. This illustrates the complexity of serological testing and is a reminder that serology is just a correlate of protection. It can be considered that this child’s antibodies might be of poor quality however this is just speculative because we did not have the opportunity to examine the antibody quality in a functional assay.

The PPV23 respondergroup was heterogenous regarding background disease and included, among others, three complement C2 deficient children, two children with congential asplenia, one child with leukemia (vaccinated with the PPV23 during sustainment therapy) and one child who had undergone stem cell transplantation.

In the PCV era it has been a matter of debate if a supplemental booster PPV23 vaccine is beneficial in the interest of broadening the serotype coverage in children with a high at risk of IPD or if it makes more sense to include repeat PCV13 doses. Our results show that at least in some high risk children PPV23 provides a good response, however the response is individual and somewhat inpredictable.

Further, it must be kept in mind that serotype-specific correlates of protection are not necessarily adequate to predict the level of clinical protection in individuals. We do, nevertheless, on the basis of our findings and others [40–43] find it beneficial to recommend PPV23 vaccination as a supplement to primary PCV vaccination.

Children experiencing rIPD are a heterogenous group and may respond insufficiently to PPV23 vaccination, therefore each child should probably be assessed individually with measurement of PNA pre- and post immunization to tailor the best programme.

A limitation of our study is the retrospective design. A prospective study adressing pneumococccal vaccine response in children with recurrent IPD could in more detail adress the specific response after and before vaccination and compare different strategies concerning revaccination. Moreover, due to the long time span of the study, the children in our cohort were exposed to different pneumococcal vaccine regimes for high risk children, which makes it difficult to compare the children and extrapolate the results directy to high risk children in 2016. Also we did not have the possibility to measure the opsonophagocytic activity of the antibodies which could have further illucidate the functional vaccine response.

We do, however, find the results important. This Danish cohort of children with rIPD is populationbased and one of the largest known cohorts to date. To our knowledge, a study of vaccination response of an unselected cohort of children with rIPD has not previously been carried out.

## Conclusion

In conclusion, we document that impaired vaccination response to PPV23 is a frequent phenomenon in this clinical narrow group of children with rIPD, while the phenomen occurred more rarely with PCV. This implies that vaccination and vaccination response children with underlying conditions should be assessed on an individual basis. Optimal pneumococcal vaccination of risk groups with combination regimes of vaccines to broaden serotype coverage seems rational. Consequently, it will be of importance to continuously evaluate the benefits of combination vaccines in different subpopulations of immune deficient children in the future, also in prospective studies.

## Additional file

Additional file 1: **Table S1.** Serotype specific vaccination response. The table contains the available rawdata concerning the serotype specific concentrations from all serological tests. Moreover the table includes data concerning time of vaccination. (DOCX 25 kb)

## Abbreviations

CVID: Common Variable Immunodeficiency
IPD: Invasive pneumococcal disease
PCV: pneumococcal conjugate vaccination
PCV13: 13-valent pneumococcal conjugate vaccine
PCV7: 7-valent pneumococcal conjugate vaccine
PNA response: serological pneumococcal antibody response.
PPV: pneumococcal polysaccharide vaccination
PPV23: 23-valent pneumococcal polysaccharide vaccine
rIPD: recurrent IPD
SAD: Selective antibody deficiency toward pneumococcal polysaccharides
TLR: Toll-like receptor
