# Supplementary material for: Serologic response to pneumococcal vaccination in children experiencing recurrent invasive pneumococcal disease
Source: BMC Infect Dis. 2018 Aug 6;18:366. doi: 10.1186/s12879-018-3267-6 (PMC6080377; doi:10.1186/s12879-018-3267-6)
Supplement: Supplementary file 1 — Table S1. Serotype specific vaccination response. The table contains the available rawdata concerning the serotype specific concentrations from all serological tests. Moreover the table includes data concerning time of vaccination. (DOCX 25 kb) [file 12879_2018_3267_MOESM1_ESM.docx]

Supplementary table 1 Serotypespecific vaccination response

| Patient | Pneumococcal vaccine | Serotype specific response | | | | | | | | | | | | | Responder (R) /  Non-responder (NR) |
| --- | --- | --- | --- | --- | --- | --- | --- | --- | --- | --- | --- | --- | --- | --- | --- |
|  |  | 1 | 3 | 4 | 5 | 6B | 7F | 9V | 14 | 18C | 19A | 19F | 23F | GG |  |
| 1 | PPV23 2010 ug/ml | 0.12 | 0.27 | 0.34 | 0.07 | 0.51 | 0.14 | 0.13 | 5.88 | 0.17 | 0.55 | 1.99 | 0.76 |  | NR |
|  | 2011 ug/ml | 0.16 | 0.03 | 0.27 | 0.10 | 0.15 | 0.08 | 0.33 | 4.66 | 0.12 | 0.49 | 1.29 | 0.44 |  |  |
| 2 | PPV23 2002 AU | low | low | low | low | low | low | low | — | — | — | — | — |  | NR |
|  | PPV23 2007 AU | 6.89 | 15.5 | 5.83 | 4.40 | 25.9 | 11.3 | 9.64 | — | — | — | — | — |  |  |
|  | PPV23 2010 AU | 13.9 | 21.1 | 10.4 | 8.08 | 14.0 | 17.3 | 13.5 | — | — | — | — | — |  |  |
|  | PPV23 2011 ug/ml | 0.17 | 0.01 | 0.08 | 0.19 | 0.08 | 0.08 | 0.13 | 0.03 | 0.06 | 0.15 | 0.30 | 0.39 |  | NR |
| 5 | PPV23 2008 AU | 20.9 |  | 67.8 |  |  | 30.2 |  | 12.5 | 43.6 |  | 38.2 |  | 31.0 | NR |
| 4 | PPV23 1998 AU | low |  | low |  |  | low |  | low | low |  | low |  | low | NR |
|  | PPV23 1999AU | low |  | low |  |  | low |  | low | low |  | low |  | low |  |
| 3 | PPV23 1992AU | low |  | low |  |  | low |  | low | low |  | low |  | low |  |
|  | PPV23 1995 AU | low |  | low |  |  | low |  | low | low |  | low |  | low | NR |
|  | PPV23 1996AU | low |  | low |  |  | low |  | low | low |  | low |  | low |  |
|  |  |  |  |  |  |  |  |  |  |  |  |  |  |  |  |
|  |  |  |  |  |  |  |  |  |  |  |  |  |  |  |  |
| 6 | PCV7 x2 2007ug/ml |  |  | 1.32 |  | 0.94 |  | 0.30 | 1.19 | 2.95 |  | 9.67 | 0.35 |  | PCV7 5/7 ok |
|  | PPV23 2008 AU | 13.4 |  | 16.7 |  |  | 14.4 |  | 5.95 | 17.7 |  | 33.7 |  | 15.0 | PPV23 NR |
| 9 | PCV7 2006 ug/ml |  |  | 0.18 |  | 0.59 |  | 0.65 | 0.84 | 0.37 |  | 1.77 | 0.27 |  | PCV7 5/7 ok |
|  | PCV7 2007 ug/ml | ---- | ---- | 1.61 | ---- | 16.6 | ---- | 1.52 | 0.83 | 0.57 | ---- | 2.50 | 0.68 |  | PCV7 R |
|  | PPV23 2008 sept AU | 14.4 | ---- | 42.8 | ---- | ---- | 32.4 | ---- | 5.68 | 25.9 | ---- | 123 | ---- | 26.7 | PPV23 NR |
|  | PPV23 2008 nov AU | 26.5 | ---- | 69.6 | ---- | ---- | 58.8 | ---- | 7.64 | 68 | ---- | 110 | ---- | 42.8 | PPV23 NR |
| 7 | PCV7 x 2 2006 ug/ml |  |  | 0.16 |  | 0.52 |  | 0.55 | 0.43 | 0.09 |  | 0.67 | 0.24 |  | PCV7 4/7 ok |
|  | PCV7 x 3 2006 ug/ml |  |  | 0.21 |  | 0.8 |  | 0.56 | 0.63 | 0.17 |  | 0.97 | 0.34 |  | PCV7 4/7 ok |
|  | PPV23 2006 AU | 10.4 | ---- | 9.87 | ---- | ---- | 2.79 | ---- | 6.65 | 2.29 | ---- | 17.8 | ---- | 6.60 | PPV23 NR |
|  | PPV23 2007 AU | 15.4 | ---- | 29.3 | ---- | ---- | 7.50 | ---- | 18.0 | 19.8 | ---- | 43.1 | ---- | 19.323 | PPV23 NR |
|  | PPV23 2009 AU | 17.9 | ---- | 56.7 | ---- | ---- | 30.5 | ---- | 17.9 | 32.1 | ---- | 102 | ---- | 34.7 | PPV23 NR |
| 12 | PCV7x2 2004 ug/ml  PPV23 2005 AU  PPV23 2011 ug/ml | 144  3.41 | 0.43 | 0.58  144  3.47 | 0.89 | 0.61  13.1  3.28 | 4.26  0.82 | 0.36  0.85 | 0.41  100  1.79 | 0.71  90  2.03 | 1.06 | 0.52  236  1.79 | 1.94 | 50.7  36.5 | PCV7 R  PPV23 R |
| 10 | PCV7 x3 2006 ug/m  PPV23 2007 AU | 20 |  | 0.82  18 |  | 0.60  14.1 |  | 1.17 | 3.47  21 | 0.88  17 |  | 0.44  32 | 0.29 |  | PCV7 R  PPV23 NR |
| 11 | PCV7 x 3 ug/ml |  |  | 0.25 |  | 0.41 |  | 0.12 | 0.49 | 0.05 |  | 0.42 | 0.52 |  | 4/7 ok |
| 15 | PPV23 2002 AU  2003 AU  2006 AU | ok  High  53.0 |  | ok  High  20.0 |  |  | High  High  33.5 |  | High  High  38.9 | High  High  - |  | High  High  52.1 |  | High  High  37,2 | PPV23 R |
| 14 | PPV23 2004 AU | 13.7 |  | 170 |  |  | 71.0 |  | 148 | 34 |  | 95.3 |  | 65.5 | PPV23 R |
| 16 | PPV23 2007 AU | 22,3  86.0  47.2 |  | 22.2  50.8  46.1 |  |  | 30.2  40.6  34.3 |  | 7.35  221  35.8 | 21.2  84.8  184 |  | 37.3  71.9  30.2 |  | 45.6  78.8  47.1 | PPV23 R |
| 17 | PPV23 2002 AU  2006 AU  2008 AU | High  30.7  15.1 |  | High  50.5  36.6 |  |  | High  42.8  48.4 |  | High  65.1  41.0 | High  16.4  15.7 |  | High  68.1  188 |  | High  41.0  38.5 | PPV23 R |
| 18 | PCV7 x2 2007 ug/ml  PPV23 2008 AU  2011 ug/ml | 14.4  26.5  1.14 | 35.8  69.6  0.7 | 58.8  32.4  0.54 | 7.64  7.32  0.17 | 29.2  68.0  1.64 | 120  110  0.27 | 27.8  42.8  1.23 | 0.77 | 0.42 | 0.21 | 0.81 | 1.38 |  | PCV7 R  PPV23 R |
| 19 | PCV7 x 3 pre ug/ml  post ug/ml |  |  | 0.11  6.73 |  | 0.22  23.5 |  | 0.06  17.5 | 0.23  14.1 | 0.04  6.54 |  | 0.21  7.93 | 0.10  43.5 |  | PCV7 R |
| 21 | PCV7 x 2 2007 AU | 19.8 | 32.1 | 19.5 | 6.31 | 17.7 | 68.2 | 22.9 |  |  |  |  |  |  | PCV7 R |
| 19 | PCV7 x 2 2003 AU | 16.8 | 46.1 | 12.1 | 86.7 | 10.7 | 57 | 27.1 |  |  |  |  |  |  | PCV7 R |
| 26 | PCV23 2001 AU | 14.4 |  | 28.2 |  |  | 137 |  | 160 | 11.6 |  | 66.6 |  | 43.7 | PPV23 R |
| 22 | PPV23 1989 AU | High |  | High |  |  | High |  | High | high |  | high |  | high | PPV23 R |
| 22  23  24  25  26 | PPV23  PPV23  PCV7 x 2, PPV23  PCV13 ug/ml  PCV7 ug/ml | High  High  1,9  1,16  1.12 | 2,1  0,9  0.7 | High  High  0,33  0.51  0.44 | 0.32  0.12  0.19 | 1,19  0.99  1.84 | High  High  0,84  0,31  0.28 | 1,18  1,16  1.34 | High  High  0,72  0,61  0.56 | High  High  0,43  0,51  0.41 | 0,37  0,36  0.24 | High  High  0,61  0,71  0.91 | 21,4  42,3  1.49 | High  High | PPV23 R  PPV23 R  PCV7 R, PPV23 R  PCV13 R  PCV7 R |
